# A proposal for analyzing the inflammatory and remodeling processes of mucosa in chronic rhinosinusitis with nasal polyposis through MRI

**DOI:** 10.1016/j.bjorl.2024.101490

**Published:** 2024-09-02

**Authors:** Débora de Carvalho Garcez, Miguel Soares Tepedino, Debora Petrungaro Migueis, Pedro Miño Vianna, Andrea Santos Dumont Costacurta, Elyzabeth Avvad Portari, Alexandre Malta da Costa Messeder, Richard Louis Voegels, Alexandre Coelho Boggi, Reginaldo Raimundo Fujita, Andrew Thamboo, Rogério Pezato

**Affiliations:** a Universidade Federal de São Paulo, Departamento de Otorrinolaringologia-Cirurgia de Cabeça e Pescoço, São Paulo, SP, Brazil; b Universidade do Estado do Rio de Janeiro, Hospital Universitário Pedro Ernesto, Rio de Janeiro, RJ, Brazil; c Universidade do Estado do Rio de Janeiro, Divisão de Rinologia e Base do Crânio, Rio de Janeiro, RJ, Brazil; d Universidade Federal Fluminense, Departamento de Otorrinolaringologia, Rio de Janeiro, RJ, Brazil; e Universidade de São Paulo, Departamento de Oftalmologia e Otorrinolaringologia, São Paulo, SP, Brazil; f University of British Columbia, Division of Rhinology, Vancouver, Canada

**Keywords:** Radiology of the paranasal sinuses, Sinusitis, Nasal polyps, Tissue remodeling, Inflammation

## Abstract

**Objectives:**

Chronic Rhinosinusitis with Nasal Polyposis (CRSwNP) is a disease characterized by chronic inflammation and tissue remodeling process. The remodeling process in nasal polyps has mainly been studied by histology analysis. However, it is limited to a polyp fragment and requires tissue removal. The present study aims to evaluate the ability of Magnetic Resonance Imaging (MRI) to depict and characterize the remodeling process in patients with CRSwNP.

**Methods:**

30 patients that met clinical diagnostic criteria for CRSwNP, without previous history of rhinosinusitis surgery were submitted to MRI scan (conventional, diffusion-weighted and DCE MRI) and compared with polyp tissue histological findings, IL-6 concentrations in the tissue and eosinophil count in the blood. The examinations were evaluated, independently, by two radiologists blinded to other radiological and histological data. The pathologist, blinded to MRI results, also compared the tissue sample from the most central and the most peripheral portion of the polypoid tissue adjacent to the floor of the nasal fossa.

**Results:**

This study demonstrated a characteristic pattern of nasal polyps, whose peripheral portions of nasal polypoid tissue are edematous, whereas the central portions in the middle meatus and in the middle and upper ethmoid are predominantly fibrotic. ADC values found in the most anterior portion of the polyps may be a marker for radiological phenotyping the remodeling process. This non-invasive analysis presented a high degree of agreement in the fibrosis and edema rating by two radiologists and the histological analysis was concordant with the MRI findings. The polyps were characterized as eosinophilic, and no relationship was found between the severity of the eosinophilic inflammatory process or concentration of IL-6 and the remodeling process.

**Conclusion:**

MRI by using T2-weighted imaging sequence and ADCs values allows tissue characterization and is an effective tool for the differentiation of edematous and fibrotic components in CRSwNP.

**Level of evidence:**

3.

## Highlights

• MRI allows the characterization of tissue remodeling in Nasal Polyps (NP).
• ADC values may be a marker for radiological phenotyping the NP remodeling process.
• There was significant agreement in the radiologist’s evaluation of edema/fibrosis.
• The MRI studies showed fibrosis in the central portion of the middle meatus.
• MRI may be a reliable tool for monitoring and guiding treatment in CRSwNP.

## Introduction

Magnetic Resonance Imaging (MRI) may identify patterns of edema, fibrosis, and infiltrates that can indicate the staging of inflammatory conditions. Chronic Rhinosinusitis with Nasal Polyposis (CRSwNP) is characterized by benign overgrowth of the rhinosinusal mucosa, which can develop polyps bilaterally,^1^ but most patients with this chronic inflammatory process do not present polyps, such as allergic rhinitis. The altered tissue remodeling process in CRSwNP^2^, ^3^, ^4^ leads to mechanical dysfunction, polyps formation, and acts as a trigger and perpetuator.^5^ The tissue remodeling is a dynamic and complex process affected by comorbidities, medications, and surgical procedures, causing temporary or permanent changes in the histological composition of the tissues.^4^ However, the knowledge of the remodeling process and its duality between edema and fibrosis remains limited, showing a more fibrous tissue in Chronic Rhinosinusitis without Nasal Polyposis (CRSsNP), in contrast to a predominantly edematous tissue in CRSwNP.^2^

The clinical expression of CRSwNP depends on the Th cell that conducts the inflammatory response. Interleukin-6 (IL-6) is a cytokine with a pathogenic role in chronic rhinosinusitis,^6^ which stimulates fibroblast proliferation and collagen production, decreasing collagen degradation in the extracellular matrix.^7^, ^8^ Moreover, IL-6 leads to an adaptative chronic inflammatory reaction, T cell recruitment and survival.^7^, ^9^ The inflammation pathway has acquired relevance for clinical practice. While eosinophils are associated with Th2 inflammation, the neutrophil and IL-6 levels are associated with non-Th2 endotypes.

Considering the relevance of endotyping the inflammatory process for the individualized treatment of CRSwNP, determining the process of tissue remodeling can optimize therapy according to the predominant tissue change, whether fibrosis or edema, as has been increasingly used in other conditions, such as Crohn’s disease^10^, ^11^, ^12^, ^13^, ^14^, ^15^ and chronic hepatitis,^16^, ^17^ contributing to precision medicine.

Computed Tomography (CT) is the imaging method most used to evaluate CRSwNP,^18^ whereby its extension is analyzed based by Lund Mackay staging system^19^, ^20^, ^21^, ^22^, ^23^ in CT and MRI.^24^ The bone characterization through CT is also important in surgical planning but does not differentiate polyps from the surrounding secretions/mucosal edema or to establish the tissue remodeling process of nasal polyps by the identification of the edematous or fibrotic’s component. Similarly, nasal endoscopy also does not evaluate tissue remodeling. Histological examination can demonstrate the remodeling process, but besides the invasive character of the procedure, the information gathered through this approach is limited to the portion of tissue collected in the biopsy and does not necessarily represent the entire tissue remodeling process of rhinosinusitis.

Conventional MRI sequences can provide morphological information in CRSwNP similarly to CT, albeit outperforming CT in tissue identification of high or hematic protein secretion/content, fibrous areas permeating the polypoid tissue. The conventional T2-weighted sequence can differentiate fibrosis and edema in other diseases.^10^ Besides, fibrosis has not been correlated to acute histological inflammation.^11^ New MRI techniques have enabled a better assessment of physiological processes and cellular metabolism of the biological tissue microenvironment, for example, the studies of angiogenesis using the perfusion technique and the analysis of cellularity by diffusion stand out.

The superior MRI tissue discrimination combined with the anatomical aspects could allow a more comprehensive evaluation of the remodeling process in CRSwNP, avoiding ionizing radiation. Sasaki et al. published on MRI of nasosinusal diseases, including permeability studies and analyzes of the Apparent Diffusion Coefficient (ADC), registering reliable sequences to identify benign and malignant lesions.^25^, ^26^ ADC can distinguish mucosal thickening from other inflammatory alterations in the maxillary sinuses and can determine some types of lesions, for example, differentiating rhabdomyosarcoma from carcinoma and lymphoma.^27^, ^28^, ^29^, ^30^, ^31^ But so far, no study on MRI of CRSwNP has been carried out. The purpose of our study is to evaluate the tissue remodeling pattern (edema/fibrosis) in CRSwNP by MRI.

## Methods

This study was approved by the Research Ethics Committee of Pedro Ernesto Universitary Hospital/UERJ and conducted in collaboration with the UNIFESP. All participants signed an informed consent form before sample collection (CAAE: 01515218.30000.5505).

Thirty patients with CRSwNP and nasal polyps exceeding the boundaries of the middle meatus bilaterally were included in this single-center hospital cross-sectional study. CRSwNP diagnosis was based on medical history, clinical examination, nasal endoscopy, and CT of the paranasal sinuses according to the European Position Paper on Rhinosinusitis and Nasal Polyps (EPOS) 2020.^32^ Individuals with severe systemic diseases, immunosuppressants use, previous history of rhinosinusitis surgery and those who did not meet the criterion of a minimum 30-day washout period for systemic or topical corticosteroids or non-steroidal anti-inflammatory drugs were excluded.

All underwent a face MRI scan. Subsequently, an endonasal endoscopic biopsy was obtained, with unilateral (randomly chosen) samples from the most anterior (peripheral) portion of the polypoid tissue and from the middle meatus central portion of the polypoid tissue. The interval between the MRI and biopsy was till 7 days. The fibrosis and edema of polypoid tissue underwent histological analysis, which was compared with MRI data.

### Qualitative assessment

The examinations were evaluated, separately, by two head and neck radiologists, each one blinded to the other radiologist and histological analysis. Each radiologist and a pathologist, blinded to MRI results, compared the image aspect of the most central portion of the polypoid tissue located in the plane of the middle meatus with the most peripheral portion of the polypoid tissue located closer to the floor of the nasal fossa, grading fibrosis and edema. Finally, the scores of both radiologists were compared, as well as the scores of each radiologist with the histopathological scores. Methods to assess polyp histology^32^, ^33^ and score edema/fibrosis of nasal samples are available in the Supplementary material.

### Quantitative assessment

ADC analyses were used to evaluate the edema and compare the peripheral and central portions of the polypoid tissue. ADC and histological findings were also compared. Eosinophil and neutrophil count, IL-6 concentrations in the tissue, and eosinophil count in the blood were applied to characterize the remodeling process and the inflammation type 2 and non-type 2, according to EPOS 2020.^32^ Eosinophil, neutrophil, and IL-6 assessments are available in the Supplementary material.

### MR imaging

MRI examinations were performed on a General Electric (GE) 1.5T Optima MR360 Advance (General Electric Healthcare, Waukesha, WI) with a 16-channel, 16-element head-and-neck coil, with a paranasal sinus protocol with T1-weighted, T2-weighted, Short Tau Inversion Recovery (STIR) and Diffusion-Weighted Imaging (DWI)-weighted sequences acquired in the coronal plane, perpendicular to the hard palate, and covering the entire length of the paranasal cavities. After intravenous contrast administration, a perfusion study was performed using the Dynamic Contrast Enhancement (DCE) technique, in addition to acquiring a post-contrast 3D-T1 weighted sequence.

### Conventional MR imaging

#### Image analysis for T2-weighted imaging

Tissue edema can be easily detected on a T2-weighted image as a hypersignal area, with greater sensitivity and specificity than other imaging modalities, including CT. Conversely, fibrosis invariably reduces the T2 signal. In addition to contributing to signal heterogeneity, the protein and/or hematic content permeating the polyps, as well as the fibrotic tissue can also reduce the T2 signal. In these cases, the examination of progressive and delayed enhancement, typical of fibrotic tissue, helped.

**Figure 1:**
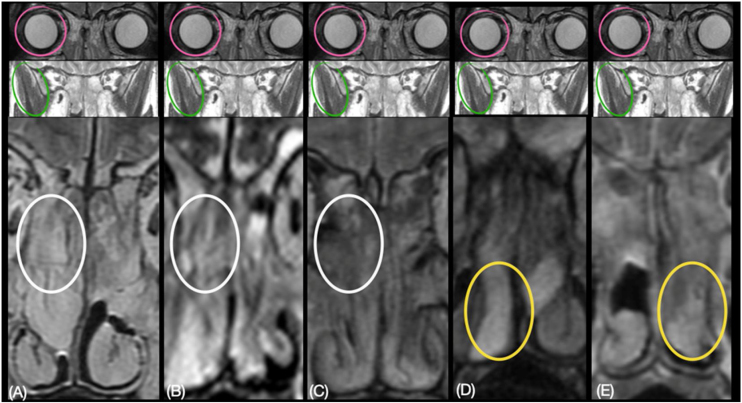
Muscle signal (green circle) and the vitreous body signal (pink circle) used as reference to score fibrosis and edema in the most peripheral portion of polypoid tissue (closer to the floor of the nasal fossa — yellow circle) and central portion (middle meatus — white circle) of polypoid tissue. For example: (A) central portion of polypoid tissue with severe edema (grade 3) and absence of fibrosis (grade 1); (B) central portion of polypoid tissue with mild edema (grade 1) and mild fibrosis (grade 1); (C) central portion of polypoid tissue with absence of edema (grade 0) and severe fibrosis (grade 2); (D) peripheral portion of polypoid tissue with severe edema (grade 3) and absence of fibrosis (grade 0); (E) peripheral portion of polypoid tissue with moderate edema (grade 2) and mild fibrosis (grade 1).

For tissue fibrosis, the parameter was the muscle signal, whereas, for tissue edema, the vitreous body signal from the eyeball was used as reference, as already done.^34^ To evaluate tissue fibrosis, the radiologists rated the most peripheral portion of polypoid tissue (closer to the floor of the nasal fossa) and central portion (middle meatus) of polypoid tissue from 0 to 2, with 0 for absence of fibrosis, 1 for mild and 2 for severe fibrosis.^34^ Similarly, to evaluate tissue edema, the radiologists rated the most peripheral and central portion of polypoid tissue from 0 to 3, with 0 for absence of edema, 1 for mild, 2 for moderate, and 3 for severe edema^34^ (Fig. 1).

#### Image analysis for diffusion-weighted and DCE MR imaging

**Figure 2:**
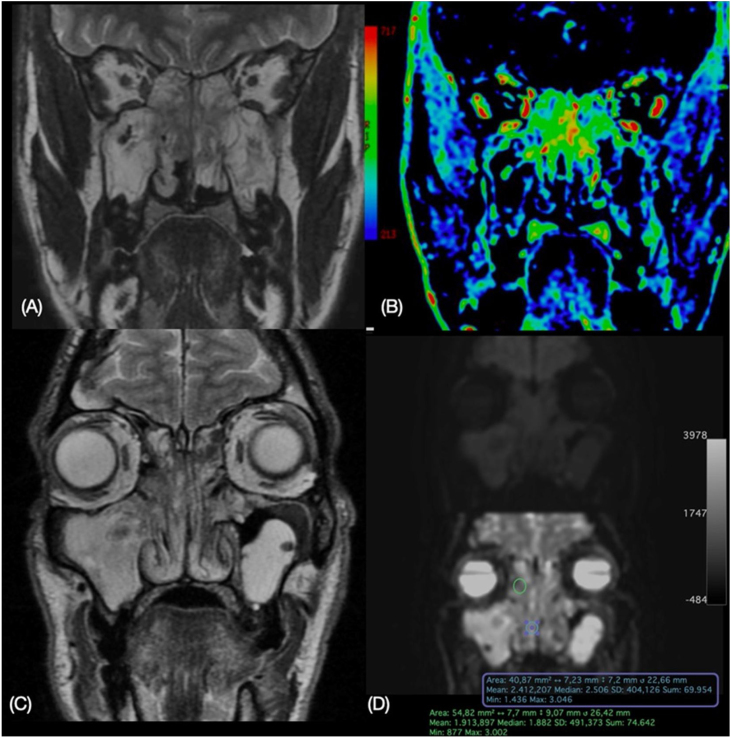
(A) T2-weighted image and (B) perfusion (PWI) color map shows increased perfusion areas in the central portion than in the peripheral portion, (C) T2-weighted image and (D) diffusion (DWI) and ADC map with values calculated by positioning the ROI in the central portion – green circle – and in the peripheral portion — blue circle.

To evaluate tissue by DWI, ADC mapping was performed to measure the degree of diffusion. Subsequently, the most representative fibrous areas, with the hypointense on T2 were selected. In these representative areas, the ADC values were calculated by manual positioning of the circular Region Of Interest (ROI) with a constant area (5 mm^2^). Similarly, ADC values were also measured in the most peripheral portions of the polyps by manual positioning of the circular ROI with 5 mm^2^, always looking for the areas with the hypointense on T2 for ROI positioning (Fig. 2).

### Statistical analysis

The results were assessed for normal distribution using the Kolmogorov–Smirnov test. The nonparametric tests Mann–Whitney, Wilcoxon and Spearman’s rank correlation were conducted. χ^2^ test was utilized to evaluate categorical variables. For all tests, a *p-*value ≤0.05 was considered significant. All statistical tests and plots were performed using smSTATA 14.0 (Stata, College Station, Texas, US).

## Results

The 30 participants’ mean age was 53 years (median: 56, range: 23‒72). There was no correlation between age and concentration of eosinophils or ADC values in the peripheral or central portion of the polyp.

From 30 patients, 13 (43%) were female and 8 (27%) presented asthma comorbidity. There was no statistical difference between the presence of asthma or gender in eosinophil count in polypoid tissue or peripheral or central ADC value. The concentration of eosinophils in the blood was higher in individuals with asthma when compared to those without asthma (*p* = 0.049). Asthma comorbidity was not correlated with edema or fibrosis on MRI or histological analysis.

### MRI findings

#### Conventional MR imaging

The results showed a high degree of agreement, approximately 90%, in the fibrosis and edema rating by the radiologists. In the MRI studies, 93.5% of patients showed some degree of fibrosis in the central portion of the middle meatus, and in contrast only 6.6% patients showed signs of fibrosis in the most peripheral portion of the polyp. A significant agreement was found in the radiologist’s evaluation of the fibrotic areas.

**Figure 3:**
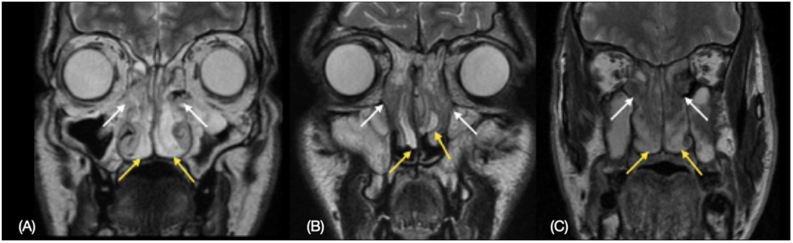
Illustration of the MRI difference between the peripheral and central regions of the nasal polyposis in T2-weighted image. (A‒C) Hyperintense on T2-weighted in the peripheral portion (yellow arrow) contrasting with areas of hypointense on T2-weighted in the central portion (white arrow).

The results also showed a pattern regarding the location of these fibrotic and edematous areas. Fibrotic areas were identified in the region of the middle meatus and in the middle and superior ethmoid bone, adjacent to the perpendicular lamina of the ethmoid bone (the most central portions of the polypoid tissue) in all cases, while the edematous areas were found in the peripheral portions of the polyps closer to the floor of the nasal fossa and in a more anterior or posterior position in the nasal cavities (Fig. 3).

### Differentiation between the central portion of the middle meatus and the peripheral portion of polypoid tissue of overall ADCs

The mean ADC values of the peripheral region were significantly higher than those of the central region of the nasal polyposis. This finding is in agreement with the T2 signal pattern observed, where the more fibrotic areas exhibited a markedly hypointense T2 signal, in contrast to the more peripheral portions of the polyp, which exhibited a markedly hyperintense T2 signal, similar to that of a liquid, with decreased cellularity and probably less collagen, therefore warranting free movement of water molecules resulting in hyperintense signal in the DWI sequence and ADC map, signaling no or minimal restricted diffusion.

**Figure 4:**
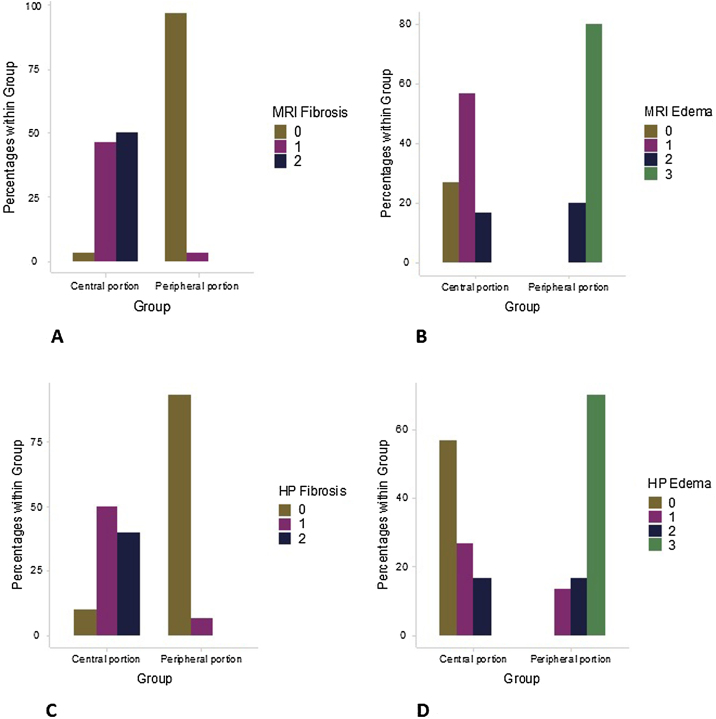
Difference in the MRI and histological rating of the tissue remodeling process between the peripheral region and the central portion of the middle meatus: MRI fibrosis (a); MRI edema (b); histological fibrosis (c); histological edema (d).

MRI-based fibrosis rating highlighted a significantly (*p* < 0.001) higher fibrosis in the central than in the peripheral region of the nasal polyposis (Fig. 4a). MRI-based edema rating highlighted a higher edema in the peripheral than in the central region of the middle meatus (Fig. 4b).

### ADC as a tissue fibrosis predictor

**Figure 5:**
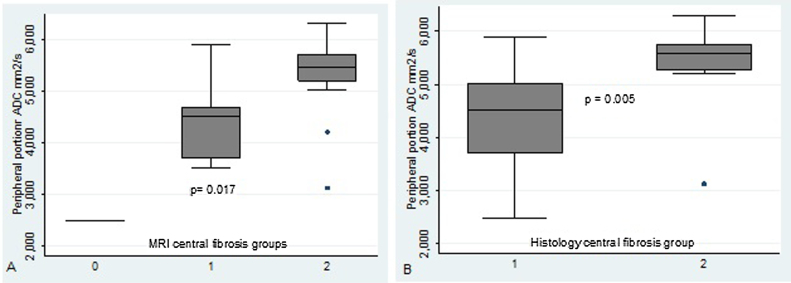
Comparison of peripheral ADC between the groups of central fibrosis, both in the fibrosis rating by the radiologists (a) and in the central fibrosis rating by the clinical pathologist (b).

The ADC values measured in the peripheral portion of the nasal polyposis in the group with severe fibrosis in the central region of the polyposis significantly differed from those of the other groups (absence or mild fibrosis) both in the groups rated by the radiologists (Fig. 5a) and in those rated by the pathologists (Fig. 5b).

### Histology

#### Comparison between the central portion of the middle meatus and the peripheral portion of nasal polyps by histology

The histological analysis was concordant with the MRI findings. A significantly (*p* < 0.001) higher degree of fibrosis was found in the central region of the middle meatus compared to the peripheral region of the nasal polyposis (Fig. 4c). Accordingly, the edema distribution was inverse, mainly found in the peripheral region and poor in the central region of the middle meatus (Fig. 4d).

#### Tissue concentration of eosinophils/neutrophils/IL-6 and the remodeling process by histology

Histological evaluation demonstrated the predominance of eosinophilic polyps among our sample, approximately 97%. No relationship was found between the severity of the eosinophilic inflammatory process and the remodeling process. Only one patient had an eosinophil count <10 3.3%, 18 had between 10 to 100 (60.1%), and 11 had a count higher than 100 eosinophils by High Powered Field (HPF) (36.6%), with a mean of 93 eosinophils (median: 46.5, range: 8–384) by HPF.

The tissue eosinophil counts, or IL-6 concentrations did not significantly differ between groups formed according to the fibrosis or edema rating based on the histological examination, regardless of the location of the biopsy, whether peripheral or central, as well as on the radiological findings.

All participants had the tissue neutrophil count <20/HPF. Fungal elements or Charcot–Leyden crystals were not evident in our sample.

## Discussion

This is the first standardized proposal of MRI analysis for CRSwNP, despite its application in other inflammatory conditions. According to a score based on previous studies,^34^ skilled radiologists reached at least 90% of agreement in the fibrosis and edema pattern despite being blind to clinical, histological, and radiological data. Besides, this study demonstrated histological heterogeneity in the same polypoid tissue as a function of tissue location and the predominance of edema in the most external (peripheral) portions of the nasal polyposis in contrast to fibrosis found inside (in the central portion). These findings challenge two common thoughts in the understanding of CRSwNP. First, histological polyp rating may be incomplete and flawed if it does not consider the location of the sample. Second, the outermost portion of the nasal polyp is predominantly edematous, in contrast to a widespread thought that given its increased exposure to antigens, airflow, and aggression the head of the polyp should be fibrous.

The identification of the fibrotic component, as well as its location in more central regions by MRI, was confirmed by the histopathological analysis. Concurrently, the absence of fibrosis in the peripheral portions of the polyp evidenced on MRI was also confirmed in the histological analysis. This imaging pattern of peripheral edema and central fibrosis suggests that polyp growth is initially related to fluid accumulation, followed by angiogenesis/vascularization to provide tissue nutrition and proliferation, and finally evolving to subepithelial fibrosis driven by chronic perpetuated repair.^35^ As the inflammatory process continues and chronicity is established damaging epithelial tight junctions in CRS patients, the peripheral edema at the head of the polyp leads the way to growth.^35^

Staging the pattern tissue remodeling seems relevant.^36^, ^37^ Chronic inflammation of the rhinosinusal mucosa may occur without nasal polyps, as in most allergic rhinitis but the development of nasal polyps does not occur without tissue remodeling process, making many experts to suggest the remodeling process in the classification of nasal disease with polyps.^38^ Although we associate tissue remodeling with fibrosis and relate inflammation with edema, edema formation also influences the extracellular matrix composition. The edema increases the volume of the polyp and consequently the surface tension applied on the endothelium of the polyp’s mucosa^5^ and these mechanical forces and changes in the shape of stem cells impact the activity of these cells and their ability to repair tissue.^39^ Studies have shown a decrease in TGF-b production in CRSwNP, associated with a decrease in TIMP-1 and an increase in metalloproteinases 9 and 7, with less collagen deposition,^2^, ^40^ which partly justifies a looser extracellular matrix that favors tissue growth.

The tissue remodeling process in CRSwNP causes histological changes in the nasal mucosa leading to water retention and edema thus creating a pressure imbalance between oncotic and hydrostatic forces that hinder the resorption of interstitial fluid associated to the inflammatory process.^3^, ^4^, ^41^ The deposition of collagen fibers in polyps restricts water molecules movement, in contrast to inflammatory edema. These histological changes match MRI findings, therefore, hypointense T2 signal areas, with high signal on the DWI sequence, and low ADC values reflect the fibrotic component, whereas T2 hyperintense signal areas, with low signal on the DWI sequence, and high ADC values indicates a predominantly edematous component.

Regarding the actual typification of the inflammatory process of CRSwNP in terms of eosinophil counts, its intensity has not been related to the remodeling process. This may partially explain why some individuals with CRSwNP do not respond to treatment even with steroids or biopharmaceuticals. Further trials are crucial for the prognostic and treatment analysis because another possible application of MRI analysis is the follow-up of anti-inflammatory and biological agents. Considering that the intensity of the inflammatory process may not be a determinant of tissue remodeling in CRSwNP, biopharmaceuticals could have their indications reassessed according to the tissue remodeling stage.

Variations in ADC values may precede changes in T2 signal intensity and thus enable us to identify changes in polypoid tissue related to disease progression or treatment, compare qualitative/quantitative evaluations, and identify the fibrotic component accurately.

Our results, albeit the small number of patients studied, suggest that the higher ADC values found in the most peripheral portion of the polyps in the group with severe fibrosis in the central portion of the middle meatus may be a marker for radiological phenotyping the remodeling process and may help predict the evolution and response to treatment. Our small sample size probably underpowered associations findings. So, a larger cohort also combined with histopathological/inflammatory analysis is certainly needed to prove that MRI is a reliable and reproducible tool in the evaluation of CRSwNP. Moreover, the predominance of eosinophilic pattern inviable the extrapolation of pathological findings observed in this sample to non-type 2 endotype. However, MRI seems to be a promising tool not only for guiding treatment decision-making in CRSwNP but also for monitoring disease activity and treatment response. The evaluation of curves from the DCE permeability study may, in future studies, compare possible differences in the inflammatory pattern of the polypoid tissue, since curves with earlier and more intense enhancement denote a more active inflammatory process, different from fibrosis, frequent in chronic inflammatory processes. Possibly, applying DCE may facilitate the CRSwNP evaluation into a dichotomized score: inflammatory vs fibrotic.

## Conclusion

In conclusion, the T2-weighted and diffusion MRI study with ADC values enables tissue characterization in CRSwNP. This study demonstrated that the peripheral portions of nasal polyp tissue are edematous, whereas the central portions (ethmoid and middle meatus) have a more fibrotic component. Further studies are necessary to demonstrate a significant correlation between the remodeling process and the severity of eosinophilic CRSwNP.

## Ethics approval and consent to participate

The study was approved by the local Research Ethics Committee, and all patients signed an informed consent form before sample collection (approval registration number: Certificate of Presentation for Ethical Consideration: 01515218.30000.5505).

## Authors’ contributions

This paper has more than seven authors who contributed substantially to the conception, design, acquisition/interpretation of data, and publishing, because participants of this study underwent clinical, radiological, histological, and surgical assessment. Then, all the listed authors meet the criteria for authorship and originality.

Conceptualization: RP. Data curation: DCG, PMV, ASDC, AMCM. Formal analysis: ACB, AT, RLV, RRF. Investigation: RRF, MST, EAP. Methodology: RP, MST, AT, RLV. Project administration: DCG, RP, MST. Resources: MST, RP, EAP, AMCM. Software: ACB, Supervision: RP, MST, AT. Validation: RLV, AT. Visualization: DPM, PMV, DCG. Writing – original draft: DPM, DCG, PMV, ASDC. Writing – review & editing: DPM, RP, RLV, MST, AT. All authors approved the final article before submission.

## Funding

None.

## Conflicts of interest

The authors declare no conflicts of interest.
