# Supplementary material for: A proposal for analyzing the inflammatory and remodeling processes of mucosa in chronic rhinosinusitis with nasal polyposis through MRI
Source: Braz J Otorhinolaryngol. 2024 Sep 2;90(6):101490. doi: 10.1016/j.bjorl.2024.101490 (PMC11414499; doi:10.1016/j.bjorl.2024.101490)
Supplement: Supplementary file 1 [file mmc1.docx]

**BJORL-D-24-00123_Supplementary Material**

**Supplementary Material**

***Nasal Tissue Samples***

Nasal tissue samples were collected by nasal biopsy from the most anterior (peripheral) portion of the polyps and from the central portion of the middle meatus. Freshly collected nasal tissues were weighed and fixed using 10% acetaldehyde and maintained for 24 hours at room temperature. The specimens were then preserved in 70% ethanol at 4°C, embedded in paraffin, and cut into 4 μm-thick sections with a microtome. Sections were then affixed onto Superfrost Plus glass slides (Menzel Glaser, Braunschweig, Germany). Once mounted, the slides were dried at 60°C for a few hours.

***Polyp Histology***

Previously fixed polyp tissue portions were stained with eosin/hematoxylin.[33] Histological examination was accessed by a pathologist under a Leica DM2000 binocular microscope at 400× magnification.

The methodology for counting eosinophils and neutrophils followed the EPOS 2020[32] to evaluate cellular predominance in the inflammatory infiltrate (Table 1). Presence of fungal elements or Charcot-Leyden crystals was conducted.

To access tissue fibrosis, the pathologist rated the most peripheral portion of polypoid tissue (closer to the floor of the nasal fossa) and central portion (middle meatus) of polypoid tissue from 0 to 2, with 0 for absence of fibrosis, 1 for mild and 2 for severe fibrosis.[33]

To quantify tissue edema, the pathologist classified the most peripheral portion of polypoid tissue (closer to the floor of the nasal fossa) and central portion (middle meatus) from 0 to 3, with 0 for absence of edema, 1 for mild, 2 for moderate and 3 for severe edema.[33]

***IL-6 measurent***

IL-6 concentrations were quantitated in protein extract from the tissue with Duo Set^®^ ELISA kits (R&D Systems, Minnesota, USA), following the manufacturer’s guidelines. Positive and negative standards and controls were prepared according to the instructions for each kit. The concentrations of cytokines were normalized by the total protein using Bradford’s method.

**Table 1** Eosinophil and neutrophil count in histological analysis.

| **Eosinophil and neutrophil count in the tissue** |
| --- |
| Eosinophils < 10/HPF |
| Eosinophils 10‒100/HPF |
| Eosinophils > 100/HPF |
| Neutrophils < 20/HPF |
| Neutrophils > 20/HPF |

HPF, High Powered Field quantification of the numbers of eosinophils or neutrophils (400×).
